# Chemical Composition and Antibacterial Effect of Clove and Thyme Essential Oils on Growth Inhibition and Biofilm Formation of *Arcobacter* spp. and Other Bacteria

**DOI:** 10.3390/antibiotics13121232

**Published:** 2024-12-20

**Authors:** Leona Hofmeisterová, Tomáš Bajer, Maciej Walczak, David Šilha

**Affiliations:** 1 Department of Biological and Biochemical Sciences, Faculty of Chemical Technology, University of Pardubice, Studentská 573, 532 10 Pardubice, Czech Republic; leona.hofmeisterova@student.upce.cz; 2 Department of Analytical Chemistry, Faculty of Chemical Technology, University of Pardubice, Studentská 573, 532 10 Pardubice, Czech Republic; tomas.bajer@upce.cz; 3 Department of Environmental Microbiology and Biotechnology, Faculty of Biological and Veterinary Sciences, Nicolaus Copernicus University in Toruń, Lwowska 1, 87-100 Toruń, Poland; maciej.walczak@umk.pl

**Keywords:** antimicrobial effect, *Arcobacter*, biofilm formation, clove, essential oil, thyme

## Abstract

**Background:** In recent years, significant resistance of microorganisms to antibiotics has been observed. A biofilm is a structure that significantly aids the survival of the microbial population and also significantly affects its resistance. **Methods:** Thyme and clove essential oils (EOs) were subjected to chemical analysis using gas chromatography coupled to mass spectrometry (GC-MS) and gas chromatography with a flame ionization detector (GC-FID). Furthermore, the antimicrobial effect of these EOs was tested in both the liquid and vapor phases using the volatilization method. The effect of the EOs on growth parameters was monitored using an RTS-8 bioreactor. However, the effect of the EOs on the biofilm formation of commonly occurring bacteria with pathogenic potential was also monitored, but for less described and yet clinically important strains of *Arcobacter* spp. **Results:** In total, 37 and 28 compounds were identified in the thyme and clove EO samples, respectively. The most common were terpenes and also derivatives of phenolic substances. Both EOs exhibited antimicrobial activity in the liquid and/or vapor phase against at least some strains. The determined antimicrobial activity of thyme and clove oil was in the range of 32–1024 µg/mL in the liquid phase and 512–1024 µg/mL in the vapor phase, respectively. The results of the antimicrobial effect are also supported by similar conclusions from monitoring growth curves using the RTS bioreactor. The effect of EOs on biofilm formation differed between strains. Biofilm formation of *Pseudomonas aeruginosa* was completely suppressed in an environment with a thyme EO concentration of 1024 µg/mL. On the other hand, increased biofilm formation was found, e.g., in an environment of low concentration (1–32 µg/mL). **Conclusions:** The potential of using natural matrices as antimicrobials or preservatives is evident. The effect of these EOs on biofilm formation, especially *Arcobacter* strains, is described for the first time.

## 1. Introduction

The emergence of resistant bacteria represents a significant and growing threat to human health [1]. Fleming warned about the possible emergence of bacterial resistance to penicillin. Humanity is now witnessing a rapid increase in the prevalence of resistant strains [2]. As a result, there has been an increase in interest in the use of natural plant products in recent years [3]. Microbial resistance is often the result of adaptation to different environmental conditions. The increase in the number of resistant bacteria in recent years can be attributed to various factors (e.g., the inappropriate use of antibiotics, inadequate infection control measures in healthcare facilities, inappropriate hygiene and sanitation procedures, etc.) [1]. Several mechanisms of antibiotic resistance are known [3]. The most common mechanisms include the inactivation of antibiotics (ATBs), often caused, for example, by the production of β-lactamases causing hydrolysis of the amide bond on the β-lactam ring. Resistance can also be related to the modification of, e.g., surface structures and receptors of microbial cells, or the ability to excrete substances that are undesirable for the cell via an efflux mechanism [3,4].

In addition, the ability to form biofilms can also be one of the mechanisms of resistance. Biofilm formation is a multi-step and complex process that includes the following: adsorption, adhesion, microcolony formation, maturation, and cell dispersion [5]. A biofilm is a three-dimensional cell structure that adheres to surfaces, enabling survival in an adverse environment [6]. Extracellular polysaccharide matrix inhibits the action of antimicrobial agents, thereby contributing significantly to resistance. Biofilms are pervasive and can be found in many aquatic and industrial systems (e.g., the food industry). Biofilm formation is well described for microorganisms of concern (e.g., *Escherichia coli*, *Listeria monocytogenes*, *Salmonella* spp., and *Campylobacter jejuni*). In addition, biofilms colonize various surfaces within households [6,7]. Biofilms in healthcare are of particular concern. Microorganisms in the form of biofilms are responsible for more than 60% of human infections. Urinary catheters, central venous catheters, indwelling stents, contact lenses, intrauterine devices, and dental chair plumbing are the most colonized medical devices by a biofilm [5].

The use of plant materials in traditional medicine has been important for many centuries. Plants represent primary healthcare for up to 80% of the population in developing countries [8]. A significant advantage of plant matrices is absolutely their minimal side effects. Antimicrobial properties are mainly attributed to quinones, phenols, alkaloids, flavonoids, terpenoids, silicon, tannins, and other secondary metabolites. However, the resulting antimicrobial activity depends on the content as well as the concentration of the sub-components [3,9]. A synergistic antimicrobial effect together with ATBs has also been described [8,10]. Essential oils (EOs) are a significant source of antimicrobial substances. These are often volatile compounds (including terpenes and terpenoids), aromatic compounds derived from phenol, or aliphatic compounds. When monitoring antimicrobial effects, it is also necessary to not neglect the substance’s influence on biofilm formation [11]. Thyme oil has a long history of medicinal use for its anti-inflammatory, antiviral, antibacterial, and antiseptic effects. One of the main compounds present in thyme essential oil is the monoterpene Thymol (2-isopropyl-5-methylphenol) [12]. The main bioactive compound of clove oil is Eugenol and its derivatives (e.g., α-Caryophyllene, Acetoeugenol, and Humulene). The significant antimicrobial, antioxidant, anti-inflammatory, and antipyretic properties of this oil have been confirmed by many studies [13].

This study is focused on monitoring the biological effect of essential oil from thyme and clove, specifically on various agents of human infections (*Pseudomonas aeruginosa*, *Escherichia coli*, *Staphylococcus aureus,* and *Enterococcus faecalis*), but also on less described bacteria of the genus *Arcobacter*. *Arcobacter* spp. are Gram-negative, spiral or slightly curved rods (0.5–2 μm long and 0.4–0.6 μm wide) [14]. Many studies confirm the pathogenicity of these little-known bacteria and their ability to cause disease in humans and animals [15,16,17], e.g., reproductive problems in livestock, mastitis, or gastric ulcers [14]. *Arcobacter* associations with human disease have been described in particular with *Arcobacter butzleri*, *Arcobacter cryaerophilus*, *Arcobacter skirrowii,* and *Arcobacter thereius* [18]. *Arcobacter butzleri* and *Arcobacter cryaerophilus* have been included on the list of microorganisms of concern to human health by the International Commission on Microbiological Specifications for Food. Genome sequencing and analysis identified nine virulence genes (*cadF*, *cj1349*, *ciaB*, *mviN*, *pldA*, *tlyA*, *hecA*, *hecB,* and *irgA*) similar to those described in *Campylobacter jejuni* [19]. Resistance of *Arcobacter* to ATBs has been described by many studies [20,21,22]. *Arcobacter* was found to be resistant to penicillins (69.3–99.2%), cephalosporins (30.5–97.4%), macrolides (10.7–39.8%), fluoroquinolones (4.3–14.0%), aminoglycosides (1.8–12.9%), and tetracyclines (0.8–7.1%) [23]. Other studies have achieved similar results [24,25,26]. Another factor in the increasing level of pathogenicity of *Arcobacter* is their ability to form a biofilm. The formation of biofilms is a common occurrence of microorganisms and is considered one of the factors in the emergence of resistant strains [21,22,27,28]. In connection with the significant described resistance of bacteria of the genus *Arcobacter*, as well as other representatives, it is desirable to search for new alternatives of substances with antimicrobial potential. Significant potential is evident in plants and other natural matrices. This study brings new findings regarding the biological effect of clove and thyme EO on planktonic cells, but also on the ability to form biofilm.

## 2. Results

### 2.1. Chemical Composition

The results of the chemical analysis of thyme and clove EOs are presented in Table 1 and Table 2, including the CAS number and the retention index of the given compound. In total, 37 and 28 compounds were identified in the thyme and clove EO samples, respectively. The identified compounds made up approximately 99.5% of the composition of the essential oil. Thyme EO was found to have the highest proportion of oxidized monoterpenes (58.29%) and monoterpenes (39.7%). Conversely, a minor amount of sesquiterpenes and diterpenes were found. The highest content was found for Thymol (44.63%), p-Cymene (26.59%), γ-Terpinene (7.01%), Carvacrol (5.97%), Linalool (4.80%), α-Pinene (2.35%), and p-Thymol (1.65%).

The highest content of phenolic derivatives (87.29%) was observed in clove EO, especially Eugenol (78.12%). The other determined significant components of this sample included sesquiterpenes (11.73%), with the highest content of (E)-β-Caryophyllene (9.72%), and α-Caryophyllene (1.66%).

### 2.2. Antimicrobial Activity

The antimicrobial potential of thyme and clove EOs is reported in Table 3 as the mean inhibition zones for *Arcobacter* strains, as well as other common bacteria (Gram-positive strains: *Staphylococcus aureus* and *Enterococcus faecalis*; Gram-negative strains: *Pseudomonas aeruginosa* and *Escherichia coli*). No inhibitory effect of thyme and clove EOs dissolved in DMSO at a concentration of 1024 µL/mL was observed. However, the antimicrobial effect of the natural EOs was observed. The inhibitory effect of EOs was in some cases even higher compared to the control ATBs (see Table 3). In general, a higher antimicrobial efficacy was observed with thyme EO. Inhibition zones of 37.3–47.5 mm and 25.0–38.0 mm were found for *Arcobacter* strains for the thyme and clove EOs, respectively. It can be stated that a lower inhibitory effect was observed against *Arcobacter defluvii* (inhibition zones of 37.3 and 25.0 mm) compared to other *Arcobacter* strains (inhibition zones up to 47.5 mm). Slightly lower antimicrobial efficiency was observed in for other Gram-negative and Gram-positive strains, with a range of inhibition zones of 21.0–41.4 mm and 7.7–17.7 mm, respectively. The weakest inhibitory effect of the tested essential oils was observed against *Pseudomonas aeruginosa* (21.0 and 7.7 mm, respectively).

The antimicrobial properties of EOs were also evaluated as minimum inhibitory concentrations (MIC) in both vapor and liquid phase, using the volatilization method (Table 4). An antimicrobial effect was observed for both EO samples, but to a different extent. However, it can be stated that a significantly higher antimicrobial effect of both samples was observed in the liquid phase. Although the samples obviously contain a number of volatile components, their antimicrobial effect or concentration is obviously insufficient to suppress the tested bacteria. The determined antimicrobial activity of thyme and clove oil was in the range of 32–1024 µg/mL in the liquid phase and 512–1024 µg/mL in the gas phase, respectively. In the liquid-phase assay, the lowest MIC value was observed in both samples for the Gram-negative *Pseudomonas aeruginosa* CCM 1961. A relatively significant antimicrobial effect (MIC 128–256 µg/mL) was also found against *Escherichia coli* CCM 2024, especially in the liquid phase. Overall, a lower inhibitory effect was observed against *Arcobacter* strains (MIC 512–1024 µg/mL) in the liquid-phase assay. However, in the vapor-phase assay, an MIC of at least 1024 µg/mL was found against all bacteria under investigation, with the exception of *Escherichia coli* CCM 2024, for which an MIC of 512 µg/mL was found.

**Figure 1:**
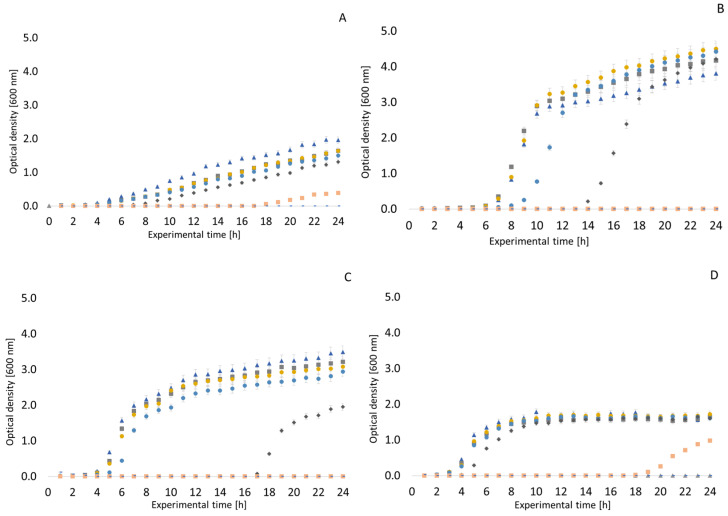
Growth curves of selected bacteria in a thyme essential oil environment. (**A**) *Pseudomonas aeruginosa* CCM 1961; (**B**) *Staphylococcus aureus* CCM 4223; (**C**) *Escherichia coli* CCM 2024; (**D**) *Enterococcus faecalis* CCM 4222. 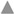 negative control; 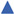 positive control; 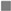 32 µg/mL; 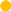 64 µg/mL; 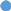 128 µg/mL; 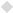 256 µg/mL; 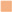 512 µg/mL; 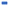 1024 µg/mL.

**Figure 2:**
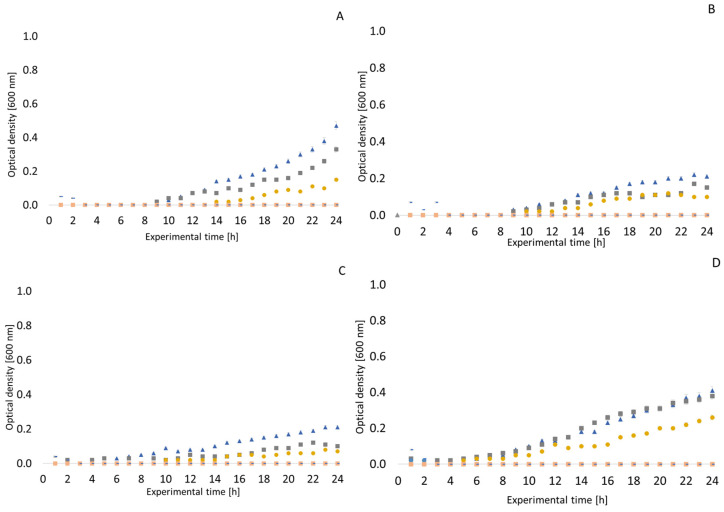
Growth curves of selected bacteria in a thyme essential oil environment. (**A**) *Arcobacter butzleri* CCUG 30484; (**B**) *Arcobacter cryaerophilus* CCM 7050; (**C**) *Arcobacter skirrowii* LMG 6621; (**D**) *Arcobacter defluvii* LMG 25694. 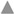 negative control; 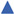 positive control; 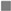 32 µg/mL; 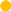 64 µg/mL; 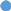 128 µg/mL; 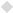 256 µg/mL; 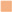 512 µg/mL; 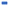 1024 µg/mL.

**Figure 3:**
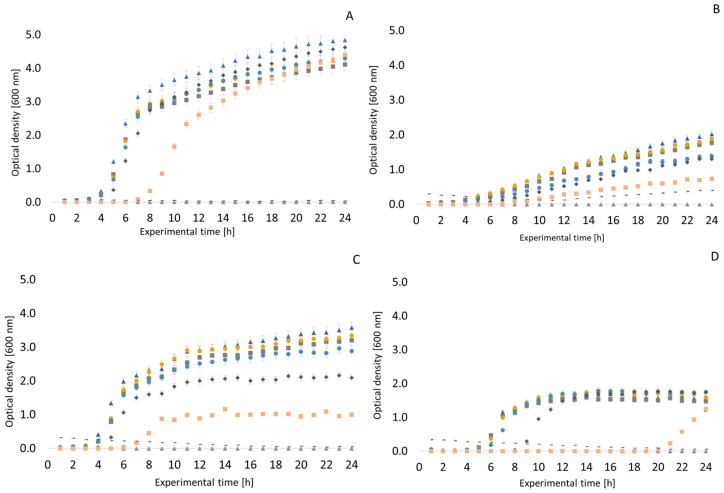
Growth curves of selected bacteria in a clove essential oil environment. (**A**) *Pseudomonas aeruginosa* CCM 1961; (**B**) *Staphylococcus aureus* CCM 4223; (**C**) *Escherichia coli* CCM 2024; (**D**) *Enterococcus faecalis* CCM 4222. 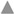 negative control; 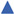 positive control; 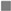 32 µg/mL; 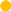 64 µg/mL; 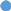 128 µg/mL; 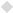 256 µg/mL; 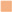 512 µg/mL; 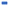 1024 µg/mL.

**Figure 4:**
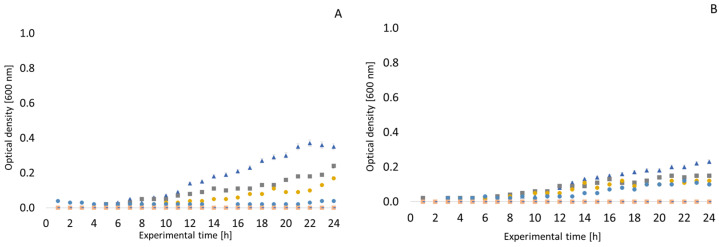

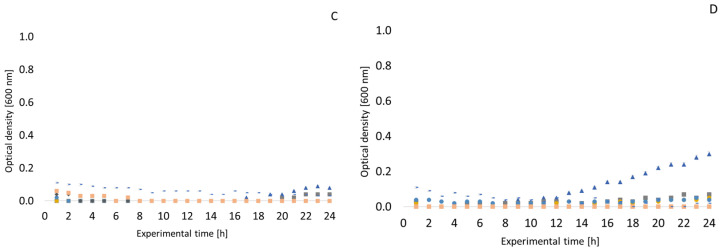
Growth curves of selected bacteria in a clove essential oil environment. (**A**) *Arcobacter butzleri* CCUG 30484; (**B**) *Arcobacter cryaerophilus* CCM 7050; (**C**) *Arcobacter skirrowii* LMG 6621; (**D**) *Arcobacter defluvii* LMG 25694. 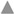 negative control; 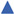 positive control; 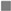 32 µg/mL; 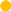 64 µg/mL; 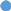 128 µg/mL; 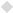 256 µg/mL; 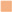 512 µg/mL; 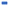 1024 µg/mL.

To better document the growth and growth curves of the monitored microorganisms in environments of thyme and clove EOs, measurements were made using an RTS-8 bioreactor. The results are presented in Figure 1, Figure 2, Figure 3 and Figure 4. In general, it can be said that the highest tested concentrations usually completely suppressed the ability of the monitored bacterial strains to survive. In the *Staphylococcus aureus* CCM 4223, no inhibitory effect of thyme EO was observed at a concentration of 32–64 µg/mL; on the contrary, there was a steeper increase in optical density (OD) compared to the experiment without the influence of EO. The highest concentrations of EO (512–1024 µg/mL) resulted in complete suppression of this pathogenic Gram-positive bacterium. In *Escherichia coli* CCM 2024, a very weak inhibitory effect of thyme EO was observed up to a concentration of 128 µg/mL. At a concentration of 256 µg/mL, the growth of this bacterium was already suppressed, which was maintained for about 17 h with a subsequent rise in OD. Higher concentrations then led to a complete suppression of *Escherichia coli* growth. An interesting inhibitory effect of thyme EO was observed on the growth of *Pseudomonas aeruginosa*. Concentrations from 512 µg/mL upwards were significantly inhibitory. A rapid rise in the OD of the *Enterococcus faecalis* CCM 4224 strain was observed approximately 3 h after the start of the experiment. With this Gram-positive bacterium, there was no noticeable inhibitory effect, even at a thyme EO concentration of 256 µg/mL; at a concentration of 512 µg/mL, after the initial suppression of growth, the OD increased from about the 19th hour of the experiment.

*Arcobacter* spp. strains had overall lower OD values than the other bacteria. For all arcobacters, it was observed that their growth is only very weakly affected by the presence of thyme EO at a concentration of 32 µg/mL and 64 µg/mL. Most strains of *Arcobacter* spp. were inhibited by thyme EO as low as 128 µg/mL. However, the exception was *Arcobacter butzleri* CCUG 30484, which was only fully inhibited at a concentration of 512 µg/mL, but at lower concentrations, the growth of this strain slowed down.

Similar growth curves to those described above were also recorded in the presence of clove EO. In general, it can be said that lower concentrations of clove oil (32, 64 and 128 µg/mL) do not have significant growth inhibitory potential. A concentration of up to 1024 µg/mL can be evaluated as the MIC. *Enterococcus faecalis* was inhibited at a concentration of 512 µg/mL until the 20th hour of exposure, but thereafter, there was a slight increase in OD (see Figure 3). Therefore, only 1024 µg/mL can be considered to be an inhibitory concentration. The growth of *Pseudomonas aeruginosa* was not completely inhibited in a medium with any concentration of clove EO. Interesting results were obtained when testing the strain *Arcobacter cryaerophilus* CCM 7050. As can be seen in Figure 4, there was a slight increase in OD between the third and sixth hour of exposure in 32 and 64 µg/mL clove oil. With *Arcobacter* strains, the inhibitory concentration of clove EO can be considered to be 512 or 1024 µg/mL. Very similar results were obtained with *Arcobacter butzleri* CCUG 30484, *Arcobacter skirrowii* LMG 6621, and *Arcobacter defluvii* LMG 25694.

### 2.3. Antibiofilm Activity

**Figure 5:**
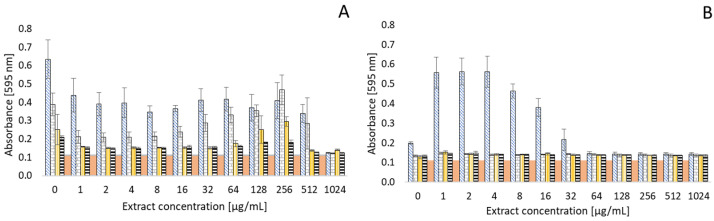
Effect of thyme EO on biofilm formation of common bacteria (**A**) and *Arcobacter* species (**B**). Data are presented as mean value of optical density (OD) ± SD. 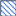 *Pseudomonas aeruginosa* CCM 1961, *Arcobacter butzleri* CCUG 30484; 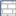 *Staphylococcus aureus* CCM 4223, *Arcobacter cryaerophilus* CCM 7050; 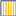 *Enterococcus faecalis* CCM 4224, *Arcobacter skirrowii* LMG 6621; 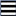 *Escherichia coli* CCM 2024, *Arcobacter defluvii* LMG 25694; 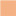 positive control.

**Figure 6:**
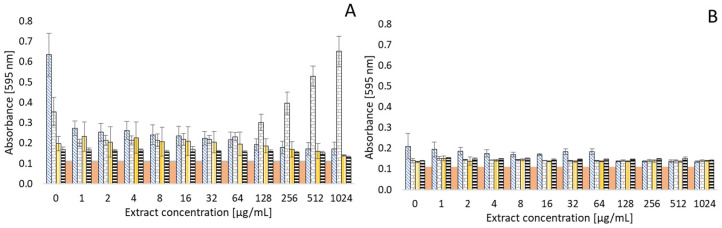
Effect of clove EO on biofilm formation of common bacteria (**A**) and *Arcobacter* species (**B**). Data are presented as mean value of optical density (OD) ± SD. 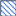 *Pseudomonas aeruginosa* CCM 1961, *Arcobacter butzleri* CCUG 30484; 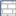 *Staphylococcus aureus* CCM 4223, *Arcobacter cryaerophilus* CCM 7050; 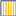 *Enterococcus faecalis* CCM 4224, *Arcobacter skirrowii* LMG 6621; 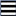 *Escherichia coli* CCM 2024, *Arcobacter defluvii* LMG 25694; 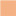 positive control.

Figure 5 and Figure 6 document the effect of thyme and clove essential oils on the biofilm formation of various bacteria. Based on preliminary tests, bacterial strains with positive biofilm formation, but with different intensities of biofilm formation, were selected for the study. In summary, a significant effect of both EOs on bacterial biofilm formation can be noted. In some strains, an inhibitory effect was observed in the presence of an EO at a certain concentration. However, on the other hand, biofilm formation was somewhat supported by the presence of an EO in other strains. The strongest biofilm formation was observed in *Pseudomonas aeruginosa*, with an initial absorbance > 0.6 in an environment without EOs. However, there was a significant decrease in biofilm formation with increasing EO concentration. Biofilm formation was almost completely suppressed in a concentration of 1024 µg/mL (OD 0.1251).

With the *Staphylococcus aureus* strain, lower concentrations of thyme and also clove EO resulted in a decrease in biofilm formation ability. However, at higher concentrations of these EOs, there was instead an increase in biofilm formation (for thyme EO at a concentration of >8 µg/mL and clove EO at >128 µg/mL, respectively). *Staphylococcus aureus* was the only strain to exhibit a significant ability to form a biofilm even in an environment with a concentration of 1024 µg/mL of clove oil (OD 0.6509). Thus, the formation was observed to be almost 50% higher than without the influence of clove EO.

Biofilm formation in *Arcobacter* strains is generally at a lower level (OD 0.1281–0.2105) than, e.g., *Pseudomonas aeruginosa* and *Staphylococcus aureus*. No such significant influence of the tested EOs on the formation of biofilms was observed for arcobacters. However, even with these bacteria, the antibiofilm effect can be evaluated in an environment with higher concentrations of EOs. In particular, a reduction in the biofilm formation of *Arcobacter butzleri* CCUG 30484 (initial OD 0.2105) can be observed in clove EO medium at a concentration of 1024 µg/mL (OD 0.1353). However, increased biofilm formation was found in the same strain in an environment of low concentrations (1–32 µg/mL) of thyme EO.

## 3. Discussion

Essential oils have been demonstrated to possess robust antimicrobial, antioxidant, anti-inflammatory, and antipyretic properties [13]. The chemical composition is always significantly influenced by the specific part of the plant used to obtain the EO. Essential oils are volatile matrices, and their vapors significantly influence antimicrobial properties [29]. Therefore, it is necessary to sufficiently investigate the composition and properties of the volatile components of the EO. In recent years, many studies have been published documenting the content of various volatile components of essential oils, and there is a need to pay more attention to their contribution to antimicrobial effects. It is clear that the volatile and non-volatile components of EO act synergistically [30,31,32,33,34,35,36].

The key component of *Thymus vulgaris* EO is the Thymol described (12 to 71%), which significantly contributes to the overall antimicrobial activity of the thyme EO [12,37]. Many studies have shown that Thymol and Carvacrol are potent antibacterial agents against both Gram-positive and Gram-negative bacteria [38,39]. The most commonly reported mechanism of antibacterial action is disruption of the bacterial membrane, leading to cell lysis. Other proposed mechanisms of antibacterial action include the inhibition of efflux pumps, the prevention of biofilm formation or damage, the inhibition of bacterial motility, and the inhibition of membrane ATPases [40,41]. With the development of nanotechnology, the encapsulation of Thymol and Carvacrol into nanocarriers has also been tested, which led to enhanced antimicrobial activity. Therefore, they are promising for use as natural antimicrobial agents or food additives [42]. The other significant compounds we identified in thyme essential oil (p-Cymene, γ-Terpinene, Carvacrol, Linalool, and α-Pinene) are considered as the main components of thyme according to previous studies [43,44,45]. Other EO components (such as β-Myrcene, Camphene, 1,8-Cineole, etc.) were determined in a total content of around 10%. Nevertheless, several chemotypes of thyme have been established based on the composition of essential oils. Chemotype describes subspecies of plants that have the same morphological characteristics but produce different amounts of chemical constituents in their EOs. In the case of thyme, chemotypes including Linalool, Borneol, geraniol, sabinene hydrate, Thymol, and Carvacrol, as well as a number of multi-component chemotypes, have been identified [46]. The chemical composition of thyme essential oil depends on geography, climate, and climatic conditions [44,47,48,49]. The claim of a different dominant component of thyme EO is also confirmed by a study of the analysis of different samples obtained in Iran. Thymol chemotype was described in some samples, while the Carvacrol chemotype was confirmed in other samples [50,51]. The composition of the oil extracted from different parts of the plant or the influence of cultivation and/or storage were also evaluated [52,53]. In addition, the composition and amount of the main components can be influenced by the type and amount of fertilizer [54,55].

Studies describing the chemical composition of EO obtained from clove leaves agree with the results presented in our study. The most abundant substance was Eugenol, followed by β-Caryophyllene [56]. The chemical composition of clove EO is significantly influenced by the geographical origin of the given matrix [57]. However, Eugenol and its derivatives (including α-Caryophyllene, Acetoeugenol, and Humulene) are reported to be the main bioactive compounds of clove oil. Earlier studies found the Eugenol content to be 76.78% [58] and 76.23% [59], which is consistent with our results (78.12%). Eugenol has been reported to have a significant bactericidal effect against a wide range of bacteria, such as *Escherichia coli*, *Pseudomonas aeruginosa*, *Listeria monocytogenes,* and *Salmonella* [60,61,62]. Previous studies suggested that the antibacterial action of Eugenol is due to the disruption of the cytoplasmic membrane, which increases its non-specific permeability [63]. However, it has been shown that the hydrophobic nature of Eugenol allows it to penetrate the lipopolysaccharide of the membranes of Gram-negative bacteria and alter the cell structure [64].

Our results using the bioreactor document the obvious antimicrobial potential of clove and thyme EO against many feared bacteria such as *Staphylococcus aureus* and *Pseudomonas aeruginosa*, but also *Enterococcus faecalis* and *Escherichia coli*. The antimicrobial effects of thyme EO against *Staphylococcus aureus* have also been confirmed by earlier studies [65,66,67]. In addition to evaluating the antimicrobial effects of EOs, the bioreactor can also be used for other experiments, e.g., *Staphylococcus aureus’* development of resistance to daptomycin was evaluated in a similar way [68]. Our results demonstrate that thyme EO inhibits the growth of *Pseudomonas aeruginosa* to a rather interesting extent, which also corresponds to other results from our study. Concentrations from 512 µg/mL upwards were significantly inhibitory, which is in line with the conclusions of an earlier study describing the antimicrobial effect of thyme EO against *Pseudomonas aeruginosa* strains isolated from meat products [69]. Another recently published study evaluated the relationship between lactoperoxidase and the shelf-life extension of meat and meat products using an RTS-8 bioreactor [70].

Significant antimicrobial effects of clove EO have been previously documented [71,72,73,74,75]. The inhibitory effect is described both against Gram-positive and Gram-negative bacteria. Although a higher resistance of Gram-negative bacteria is usually reported in connection with the construction of the cell wall [76,77], this cannot be confirmed for the results obtained in our study. However, especially if the EO is dissolved in, e.g., methanol, its lipophilic properties increase, which leads to an increase in the permeability of the cell wall. MICs of 0.64 mg/mL and 0.52 mg/mL against *Escherichia coli* and *Staphylococcus aureus*, respectively, were reported for clove oil [78]. Another study reported concentrations of 2.5 µL/mL and 5 µL/mL as the MIC and MBC, respectively, against MRSA [79]. Eugenol contributes significantly to the antimicrobial effect of clove EO [80]. Eugenol is also the dominant component of this EO, but is also found in other natural matrices such as cinnamon leaf, pepper, or *Ocinum sanctum* [81].

Furthermore, the minimum inhibitory concentrations (MICs) of the EOs were determined, both in the liquid and vapor phase, using a previously described volatilization method [82,83]. However, many previous studies have addressed the antimicrobial effect of thyme oil on many microorganisms in a liquid-phase assay. For example, a study by Sateriale et al. reports thyme EO concentrations of 20 and 100 µL/mL as the MIC and minimum bactericidal concentration (MBC) against *Salmonella* Typhimurium ST1, respectively. The MIC value is even only about 50% lower compared to the MIC found for gentamicin. A similarly interesting antimicrobial effect was also confirmed against *Bacillus cereus* BC3 [84]. In the past, a synergistic effect of thyme EO in combination with nisin has even been described against *Escherichia coli* O157:H7 in minced beef during refrigerated storage [85]. Furthermore, the enhanced antimicrobial effect of nano-emulsified thyme oil with sodium caseinate and lecithin was also described. In this case, a stronger inhibitory effect against *Escherichia coli* O157:H7 and *Listeria monocytogenes* was described, especially in the initiation phases of growth. The results even suggested the potential of using emulsified thyme EO as a food preservative. Thyme EO and Thymol are generally recognized by many studies as bio-preservatives in the meat industry [86]. The antimicrobial ability of thyme EO is due to the synergistic combination of individual components, but the highest inhibitory effect is described for Thymol [87]. Thymol has even been described as an effective substance for controlling the growth of enterotoxigenic strains of *Escherichia coli* [88]. To our knowledge, thyme and clove EOs in the vapor phase have not been evaluated so far, especially against *Arcobacter* strains. The results are therefore difficult to compare with previously published data. Antimicrobial effect of various natural EOs has also been demonstrated in earlier studies. It has been described that bergamot EO provides a strong inhibitory effect on *Arcobacter butzleri*. To a lesser extent, the antimicrobial effect of lemon and orange oil has also been described [89].

EOs are also traditionally used for inhalation purposes to treat respiratory tract infections. The results of testing the antimicrobial activity in the vapor phase can be very useful for understanding the effects of EOs in the respiratory tract [90]. Another study described the efficacy of EO vapors from lemongrass and geranium against Methicillin-resistant *Staphylococcus aureus* (MRSA) and vancomycin-resistant enterococci. After 20 h of exposure in a closed box, a 38% reduction in MRSA growth was observed. The number of airborne bacteria in a closed room was also reduced by 89% after 15 h of exposure. These results suggest the possibility of using EO for air disinfection [91]. The antimicrobial effects of EO in the vapor phase could also be used in the food industry. The effect of gaseous EO obtained from *Citrus limon* leaves against *Listeria monocytogenes* strains was evaluated [92]. The significant antimicrobial effect of EO from cinnamon and cloves in the vapor phase was also confirmed. In the future, these substances could be used in the food packaging process. This could create a protective atmosphere with minimal organoleptic changes in the packaged food [93]. This assumption is also confirmed by other studies [94,95,96].

It is important to monitor the growth parameters of bacteria in the EO environment. For this reason, we included monitoring the influence of the given samples in the bioreactor. Unfortunately, this is not described in many studies. Nevertheless, we can find some studies in the literature documenting similar conclusions. A study on the antimicrobial effects of clove EO on *Staphylococcus aureus* by Xu et al. reported entering the logarithmic phase after only two hours of exposure to lower concentrations of the oil [59]. The authors of this earlier study also reported a similar MIC value [54]. The antimicrobial effect of clove EO on *Staphylococcus aureus* and *Escherichia coli* has also been demonstrated in other studies [59,97]. Other studies also evaluated the inhibitory effects of clove EO on different strains of *Escherichia coli* [98], with conclusions similar to those of our study.

Biofilm formation is a factor in microbial virulence, and also one that significantly affects microbial resistance [99,100,101,102,103]. Strains with positive biofilm formation are considered clinically significant [5,104,105,106]. In summary, a significant effect of both EOs on bacterial biofilm formation can be noted. According to our results, it is evident that some EOs can inhibit the growth of some bacteria, but others can support biofilm formation. On the basis of an earlier study, it can be stated that the production of the biofilm of *Arcobacter* strains can be influenced, for example, by hydrolates formed during the extraction of essential oils (lavender, bay leaf, and fennel) [107]. In the case of the results of our study, a decrease in the biofilm formation ability of *Staphylococcus aureus* was observed in environments with lower concentrations of thyme and also clove EO. However, at higher concentrations of these EOs, there was instead an increase in biofilm formation (for thyme EO at a concentration of > 8 µg/mL and clove EO at > 128 µg/mL, respectively). This can be explained by the increased stress acting on the cells, which already limits the survival of the planktonic forms of the cells and immediately transitions to a “safer” biofilm structure. The ability to stimulate biofilm production at lower concentrations of various essential oils has also been supported by other studies [108,109]. Microorganisms form biofilm structures as part of a defense strategy (reduction in metabolic reactions, increase in oxidative stress, etc.). The protection of soil bacteria in life cycles is also described, where biofilm formation plays a key role in drought resistance [110,111,112,113]. The effect of Thymol on the formation of a *Staphylococcus aureus* biofilm has also been demonstrated [114]. The results of further studies confirm the possibility of stimulating biofilm formation in the environment of sub-inhibitory concentrations of natural substances. It was found that lower concentrations of cinnamon essential oil support the biofilm formation of *Staphylococcus epidermidis*. Subsequently, *icaA* expression analysis was performed using *q*PCR. Overexpression of *icaA* was observed in strains exposed to the studied concentrations of cinnamon EO [115]. In addition, thyme essential oil was also tested to suppress the biofilm formation of MRSA strains. The results confirmed the significant antibiofilm effect of thyme EO [116].

In the past, the influence of other essential oils on biofilm formation was also confirmed [117]. A significant effect of *Lippia origanoides* [118], *Ocimum gratissimum* L. [119], or *Lavandula angustifolia* [120] essential oils on *Escherichia coli* and *Staphylococcus aureus* biofilm formation have been reported. Further, the antibiofilm activity of essential oils obtained from oregano and sage was tested against *Streptococcus pyogenes* strains. The inhibition of biofilm formation was confirmed for both EOs at a concentration of 0.5 mg/mL. In contrast, penicillin G already inhibited biofilm formation at a concentration of 0.008 µg/mL [121]. Conversely, EO isolated from *Laurus nobilis* exhibited a promising ability to eradicate the biofilms of a wide spectrum of Gram-negative and Gram-positive strains [122]. An earlier study also evaluated the use of natural-based antimicrobials to remove and/or reduce an *Aeromonas hydrophila* biofilm formed on stainless steel surfaces. The results showed a significant reduction in the amount of biofilm after the application of essential oils from *Thymus vulgaris* and *Cymbopogon citratus* [123]. In the past, the effect of essential oils on yeast biofilm activity was also tested. *Mentha piperita* L. essential oil at a concentration of 2 μL/mL has been shown to inhibit *Candida albicans* and *Candida dubliniensis* biofilm formation [124].

## 4. Materials and Methods

### 4.1. Essential Oils and Sample Preparation

The following essential oils were purchased from Merck Life Science (Prague, Czech Republic): clove essential oil (C8392; *Eugenia* spp., *Myrtaceae* family) and thyme oil (W306509; *Thymus vulgaris* and/or *Thymus zygis*, *Lamiaceae* family). Since the exact chemical composition of the given essential oils was not available, a chemical analysis was performed. A volume of 2 µL of EO was dissolved in dimethylsulfoxide (DMSO) to obtain a stock solution of each essential oil at a concentration of 102,400 µg/mL.

### 4.2. Bacterial Strains

Bacterial strains used for microbiological testing were obtained from the Czech Collection of Microorganisms (CCM, Brno, Czech Republic), the Culture Collection University of Gothenburg (CCUG, Gothenburg, Sweden), and the Belgian Co-ordinated Collections of Microorganisms (LMG, Ghent, Belgium). Strains were aerobically cultured on Tryptone Soya agar (TSA, HiMedia, Mumbai, India) for 48 h at 30 °C (*Arcobacter* spp.) or 37 °C (other bacteria). Subsequently, a cell suspension was prepared in a physiological solution corresponding to level 0.5 of the McFarland basic scale (10^8^ CFU/mL). The cell suspension thus prepared was diluted to a cell density suitable for the given experiment.

### 4.3. Antimicrobial Testing

The disk diffusion method was used for basic verification of the sensitivity of the strains to the samples. A freshly prepared cell suspension with a cell density of 10^8^ CFU/mL was spread on Mueller–Hinton agar (Himedia, Thane, India) using a sterile cotton swab. Sterile disks 6 mm in diameter (Oxoid Ltd., Hampshire, UK) were impregnated with 8 μL of the sample (EOs or EOs dissolved in DMSO) based on our preliminary tests. After cultivation under optimal conditions, the diameter of the inhibition zone was evaluated using a BACMED 6iG2 automatic reader and analyzer (Aspiag, Litomyšl, Czech Republic). Commercial antibiotic disks (Sigma-Aldrich, St. Louis, MO, USA) were used as a reference standard: ampicillin (10 μg), clindamycin (2 μg), and erythromycin (15 μg). Each experiment was performed at least three times, and the inhibition zones shown are expressed as the mean of the inhibition zones, including the standard deviation.

The antimicrobial effect of EOs in the liquid and vapor phase was tested by the broth microdilution volatilization method [82]. Experiments were performed in 96-well microtiter plates with a well volume of 400 µL. For the first part of the testing (the vapor-phase assay), 30 µL of Mueller–Hinton agar was pipetted onto each lid flange and, after solidification, 5 µL of the cell suspension at a concentration of 10^8^ CFU/mL was inoculated. In the second part (the liquid-phase assay), 90 μL of buffered Mueller–Hinton broth (Himedia, India) was pipetted into each test well. A sample of EOs dissolved in DMSO was subsequently diluted twofold in broth, starting at a concentration of 1024 µg/mL. The plates were then inoculated with 10 µL of cell suspension at a concentration of (10^8^ CFU/mL). Wells containing inoculated and uninoculated broth were prepared as controls for culture vitality or broth sterility. Edge wells were excluded from testing to prevent a false edge effect. Due to the need to close the plate tightly and reduce evaporation of the sample, a wooden plate of the same format was placed on the lid of the plate and clamped. After cultivation under optimal conditions (30 or 37 °C for 24 h, aerobic atmosphere), minimal inhibitory concentrations (MICs) were assessed visually after staining for the presence of metabolically active cells with MTT dye (Thiazolyl Blue Tetrazolium Bromide; Sigma-Aldrich, St. Louis, MO, USA) showing a change in color from yellow to purple (compared to the control well) in both broth and agar. MIC values (µg/mL) were determined as the lowest concentration inhibiting bacterial growth compared to growth in a well without the presence of the target substance. All experiments were performed at least three times. The calculated standard deviations of the measurements are not shown in Table 4, but these values did not exceed 5% in any experiment.

### 4.4. Growth Parameters in EOs Environment

Bacterial growth in the presence of EOs of different concentrations was evaluated using an RTS-8 Multi-channel Bioreactor (Biosan, Riga, Latvia). Bacteria were cultured in special 50 mL centrifuge tubes (TubeSpin^®^, Biosan, Latvia) filled with 10 mL BHI broth with EOs to obtain final sample concentrations of 32–1024 µg/mL. Bacterial suspension was subsequently added to obtain a final cell concentration of 10^7^ CFU/mL. BHI broth with bacterial culture was used as a positive control for growth, and pure BHI broth was used as a negative control for the sterility of the medium. Cultivation in the bioreactor took place at the optimal temperature for the given microorganisms (30 or 37 °C) at 500 rpm for 24 h. The OD of the culture was measured automatically at 30 min intervals at 600 nm. All experiments were performed at least two times. Results are expressed as mean OD, with standard deviation indicated.

### 4.5. Biofilm Formation Testing

Biofilm formation in the presence of clove and thyme EOs was monitored in flat-bottomed microtiter plates (SPL Life Sciences, Pocheon-si, Republic of Korea) as previously described [105]. Briefly, the twofold dilutions of EOs were prepared in brain heart infusion (BHI, Himedia, India) to obtain a final concentration ranging from 0 to 1024 µg/mL in the wells after the addition of bacterial culture at a cell density of 10^8^ CFU/mL. After incubation under optimal conditions, the microtiter plate was washed with sterile distilled water and dried. Biofilm fixation was performed with 2% sodium acetate (15 min), and attached cells were stained with 100 µL of 1% crystal violet (Sigma-Aldrich, St. Louis, MO, USA). After 15 min of staining, the plate was repeatedly washed and dried. Thereafter, the biofilm-associated violet was solubilized with 96% ethanol, and the absorbance of the solution was measured in a new plate at 595 nm (Infinite M200, Tecan, Männedorf, Switzerland). The biofilm formation just in the presence of BHI is represented by a red area in the figures. There were 8 wells in each experiment, and the experiments were independently repeated 3 times. The results are expressed as mean OD with standard deviation indicated.

### 4.6. GC-MS Analysis

The essential oils were diluted with n-hexane (1:100) and analyzed by gas chromatography–mass spectrometry. Analyses were performed using a GC 2010 gas chromatograph equipped with a QP 2010 Plus mass spectrometer (both from Shimadzu, Kyoto, Japan) with a Combi Pal autosampler (CTC Analytics, AC, Zwingen, Switzerland). An amount of 1 μL of diluted sample was injected at a temperature of 200 °C with a split ratio of 1:10. Separation of the compounds was performed in an LN-5 Sil MS column (30 m × 0.25 mm × 0.25 μm) from Chromservis (Prague, Czech Republic). The carrier gas was helium 5.0 (Linde Gas a.s., Prague, Czech Republic) at a linear velocity of 30 cm/s. The temperature program was as follows: initial temperature was set to 40 °C (3 min) and then gradually increased to 250 °C at 3 °C/min. The temperature of the interface and ion source was set to 200 °C. The ionization of the substances was carried out by electron ionization at 70 eV. Mass spectra were scanned over the range of *m*/*z* 35-400.

Identification of the separated compounds was performed based on the measured mass spectra using the mass spectra libraries NIST 11 (the National Institute of Standards and Technology’s Mass Spectral Library) and FFNSC2 (Flavor and Fragrance Natural and Synthetic Compounds). Verification was performed by comparing the calculated retention indices with data from the literature. The retention indices of the separated compounds were determined experimentally by the analysis of a homologous series of n-alkanes under the same conditions as the samples.

### 4.7. GC-FID Analysis

GC-FID analyses of obtained essential oils were carried out using a GC 2010 gas chromatograph with a flame ionization detector (Shimadzu, Kyoto, Japan) and Combi Pal Autosampler (CTC Analytics, AG, Zwingen, Switzerland). The GC-FID conditions were the same as for GC-MS analysis. The injector temperature was set to 200 °C and the detector temperature to 270 °C. An amount of 1 µL of extract was injected with a split ratio of 1:50. The identification of chemical compounds was performed using experimental results of retention indices, which were calculated according to the van den Dool and Kratz method, using n-alkanes as external [125]. Calculated retention indices were additionally compared with retention indices of the identified compounds from mass spectra analysis.

### 4.8. Statistical Analysis

The obtained values were statistically evaluated using Microsoft Office 365 (Microsoft, Redmond, WA, USA) and Statistica 12 (StatSoft, Tulsa, OK, USA). Extreme values were tested with the Dean–Dixon test and all remoteness values were excluded with 95% probability. Mean and standard deviation were determined from the remaining values. A possible source of error resulting from insufficient dye washing in biofilm staining that leads to increased absorbance was also considered. Similarly, absorbance values that were too high compared to other measured values were excluded. Statistical significance was determined by chi-squared test. A *p* value of <0.05 was considered to be statistically significant. The calculated standard deviations of the measurements are not shown in Table 4, but these values did not exceed 5% in any experiment.

## 5. Conclusions

In this study, both the antimicrobial effect and the effect on biofilm formation of thyme and clove essential oils on strains of *Staphylococcus aureus*, *Pseudomonas aeruginosa*, *Escherichia coli,* and *Enterococcus faecalis,* and especially on strains of *Arcobacter* spp., were evaluated. To the best of our knowledge, there has not been much information in the literature about the antimicrobial effect of thyme and clove EO in both the vapor and liquid phases. In connection with the established antimicrobial effect and the chemical composition of EOs, the potential of using these matrices as natural antimicrobials or preservatives is evident. The effect of these EOs on biofilm formation, especially *Arcobacter* strains, is described for the first time. Of course, it is desirable to verify the results also in the case of strains isolated from the environment, which could be a further direction of research work. After verifying the effect of thyme and clove EOs on biofilm formation, it would also be useful to focus on monitoring biofilm formation under other conditions that simulate specific environments.

## Figures and Tables

**Table 1:** Chemical composition of thyme essential oil.

Compound	CAS Number	Retention Index	Peak Area (%)
**Monoterpenes**			
Tricyclene	508-32-7	919	0.01
α-Thujene	2867-05-2	924	0.01
α-Pinene	80-56-8	930	2.35
α-Fenchene	471-84-1	945	0.01
Camphene	79-92-5	947	0.67
β-Pinene	127-91-3	975	0.07
β-Myrcene	123-35-3	990	0.97
Pseudolimonene	499-97-8	1003	0.04
α-Terpinene	99-86-5	1017	0.01
m-Cymene	535-77-3	1020	0.10
Carvomenthene	5502-88-5	1022	0.02
p-Cymene	99-87-6	1027	26.59
Limonene	138-86-3	1029	0.08
o-Cymene	527-84-4	1035	0.01
(E)-β-Ocimene	3779-61-1	1047	0.06
γ-Terpinene	99-85-4	1059	7.01
p-Thymol	3228-02-2	1307	1.65
2-Methyl-6-propylphenol	89-72-5	1320	0.04
Sum (%)			39.7
**Oxidated monoterpenes**			
1,8-Cineole	470-82-6	1032	0.71
(Z)-Linalool furanoxide	5989-33-3	1072	0.01
(E)-Linalool furanoxide	34995-77-2	1088	0.01
Fenchone	1195-79-5	1090	0.01
Linalool	78-70-6	1104	4.80
Plinol A or B or C	4028-60-8	1130	0.04
Camphor	76-22-2	1150	0.92
Isoborneol	124-76-5	1166	0.60
Borneol	507-70-0	1175	0.35
4-Terpineol	562-74-3	1183	0.24
Thymol	89-83-8	1291	44.63
Carvacrol	499-75-2	1301	5.97
Sum (%)			58.29
**Sesquiterpenes**			
α-Copaene	3856-25-5	1372	0.01
(E)-β-Caryophyllene	87-44-5	1416	0.99
α-Caryophyllene	6753-98-6	1453	0.05
Sum (%)			1.05
**Oxidated sesquiterpenes**			
Caryophyllene oxide	1139-30-6	1580	0.37
Humulene epoxide II	19888-34-7	1609	0.02
Sum (%)			0.39
**Diterpenes**			
m-Camphorene	20016-73-3	1945	0.01
Sum (%)			0.01
**Total Peak Area (%)**			**99.44**

**Table 2:** Chemical composition of clove essential oil.

Compound	CAS Number	Retention Index	Peak Area (%)
**Sesquiterpenes**			
α-Cubebene	17699-14-8	1344	0.01
α-Copaene	3856-25-5	1373	0.01
β-Elemene	515-13-9	1387	0.01
(Z)-β-Caryophyllene	118-65-0	1400	0.02
(E)-β-Caryophyllene	87-44-5	1417	9.72
α-Caryophyllene	6753-98-6	1453	1.66
Alloaromadendrene	25246-27-9	1456	0.01
γ-Muurolene	30021-74-0	1472	0.01
β-Selinene	17066-67-0	1486	0.01
α-Selinene	473-13-2	1493	0.01
α-Muurolene	10208-80-7	1496	0.01
(E,E)-α-Farnesene	502-61-4	1504	0.01
α-Bulnesene	3691-11-0	1506	0.01
γ-Cadinene	39029-41-9	1510	0.03
δ-Cadinene	483-76-1	1516	0.18
α-Calacorene	21391-99-1	1540	0.02
Sum (%)			11.73
**Oxidated sesquiterpenes**			
Caryolan-8-ol	178737-45-6	1575	0.01
Caryophyllene oxide	1139-30-6	1580	0.53
Humulene epoxide II	19888-34-7	1608	0.06
Sum (%)			0.60
**Phenolic derivates**			
Chavicol	501-92-8	1261	0.01
Eugenol	97-53-0	1363	78.12
Dihydroeugenol	2785-87-7	1367	0.08
Isovanilin	621-59-0	1402	0.01
Methyleugenol	93-15-2	1405	0.04
Eugenyl acetate	93-28-7	1522	9.00
4-Hydroxy-2-methoxycinnamaldehyde	127321-19-1	1736	0.01
(E)-Coniferyl alcohol	32811-40-8	1741	0.01
Coniferyl alcohol diacetate	38209-46-0	2093	0.01
Sum (%)			87.29
**Total Peak Area (%)**			**99.62**

**Table 3:** Antimicrobial activity of clove and thyme essential oils. Results are expressed as mean of inhibition zones in mm (including disk 6 mm in diameter ± standard deviation), *n* = 5.

	Thyme		Clove		AMP	ERY	CLI
	EO	EO_DMSO_	EO	EO_DMSO_			
***Arcobacter* strains**							
*Arcobacter butzleri* CCUG 30484	47.5 ± 1.5	6.0 ± 0	29.0 ± 1.3	6.0 ± 0	14.7 ± 1.9	37.7 ± 1.2	23.7 ± 2.5
*Arcobacter cryaerophilus* CCM 7050	45.3 ± 2.6	6.0 ± 0	38.0 ± 1.8	6.0 ± 0	12.7 ± 1.2	29.3 ± 3.9	21.3 ± 0.5
*Arcobacter skirrowii* LMG 6621	46.3 ± 1.5	6.0 ± 0	32.7 ± 1.8	6.0 ± 0	13.0 ± 2.2	33.7 ± 2.4	24.0 ± 1.6
*Arcobacter defluvii* LMG 25694	37.3 ± 2.9	6.0 ± 0	25.0 ± 1.24	6.0 ± 0	11.7 ± 0.9	35.7 ± 1.2	15.7 ± 1.2
**Other bacteria**							
*Staphylococcus aureus* CCM 4223	41.4 ± 3.7	6.0 ± 0	15.8 ± 1.3	6.0 ± 0	22.3 ± 1.2	30.3 ± 0.9	28.0 ± 0.8
*Enterococcus faecalis* CCM 4224	36 ± 0.30	6.0 ± 0	15 ± 0.11	6.0 ± 0	11.3 ± 0.5	16.0 ± 0.8	10.7 ± 1.2
*Pseudomonas aeruginosa* CCM 1961	21.0 ± 2.64	6.0 ± 0	7.7 ± 1.1	6.0 ± 0	6.0 ± 0	6.0 ± 0	6.0 ± 0
*Escherichia coli* CCM 2024	37.8 ± 6.38	6.0 ± 0	17.7 ± 0.94	6.0 ± 0	6.0 ± 0	11.0 ± 0.8	6.0 ± 0

EO—essential oil; EO_DMSO_—essential oil dissolved in DMSO (1024 µg/mL); AMP—ampicillin; CLI—clindamycin; ERY—erythromycin.

**Table 4:** Minimum inhibitory concentrations of EOs and ATBs against *Arcobacter* and other bacteria. Results are expressed as mean of MICs (µg/mL), n = 3.

	Thyme		Clove		AMP	ERY	CLI
	Vapor-PA	Liquid-PA	Vapor-PA	Liquid-PA	Liquid-PA	Liquid-PA	Liquid-PA
***Arcobacter* strains**							
*Arcobacter butzleri* CCUG 30484	>1024	512	1024	1024	16	0.5	64
*Arcobacter cryaerophilus* CCM 7050	>1024	1024	1024	512	4	2	64
*Arcobacter skirrowii* LMG 6621	>1024	1024	1024	512	16	0.25	4
*Arcobacter defluvii* LMG 25694	>1024	512	1024	512	4	1	4
**Other bacteria**							
*Staphylococcus aureus* CCM 4223	1024	256	>1024	512	4	1	8
*Enterococcus faecalis* CCM 4224	1024	256	>1024	256	1	2	16
*Pseudomonas aeruginosa* CCM 1961	>1024	32	>1024	32	2	2	64
*Escherichia coli* CCM 2024	512	128	>1024	256	16	2	16

AMP—ampicillin; CLI—clindamycin; ERY—erythromycin; PA—phase assay; EOs—essential oils; ATBs—antibiotics.

## Data Availability

The original contributions presented in this study are included in the article. Further inquiries can be directed to the corresponding author.
